# Activation of LXRs Reduces Oxysterol Lipotoxicity in RPE Cells by Promoting Mitochondrial Function

**DOI:** 10.3390/nu14122473

**Published:** 2022-06-15

**Authors:** Lirong Xie, Qing Gu, Xingwei Wu, Lili Yin

**Affiliations:** 1 Department of Ophthalmology, Shanghai General Hospital (Shanghai First People’s Hospital), Shanghai Jiao Tong University School of Medicine, Shanghai 200080, China; xielirong@alumni.sjtu.edu.cn (L.X.); guqing6823@163.com (Q.G.); wuxingwei2010@tom.com (X.W.); 2 Shanghai Key Laboratory of Fundus Disease, Shanghai 200080, China; 3 Department of Ophthalmology, Shanghai Fourth People’s Hospital Affiliated to Tongji University, Shanghai 200434, China

**Keywords:** liver X receptors, lipid metabolism, lipotoxicity, mitochondrial function, age-related macular degeneration

## Abstract

Effective treatments for age-related macular degeneration (AMD), the most prevalent neurodegenerative form of blindness in older adults, are lacking. Genome-wide association studies have identified lipid metabolism and inflammation as AMD-associated pathogenic changes. Liver X receptors (LXRs) play a critical role in intracellular homeostases, such as lipid metabolism, glucose homeostasis, inflammation, and mitochondrial function. However, its specific role in AMD and its underlying molecular mechanisms remain unknown. In this study, we investigated the effects of lipotoxicity in human retinal pigmental epithelial (ARPE-19) cells and evaluated how LXRs reduce 7-ketocholesterol (7KCh) lipotoxicity in RPE cells using models, both in vivo and in vitro. A decrease in oxidative lipid accumulation was observed in mouse retinas following the activation of the LXRs; this result was also confirmed in cell experiments. At the same time, LXRs activation reduced RPE cell apoptosis induced by oxysterols. We found that oxysterols decreased the mitochondrial membrane potential in ARPE-19 cells, while LXR agonists counteracted these effects. In cultured ARPE-19 cells, activating LXRs reduced p62, mTOR, and LC3I/II levels, and the knockdown of LXRs elevated the expression of these proteins, indicating that activating LXRs could boost mitophagy. The findings of this study suggest LXR-active pharmaceuticals as a potential therapeutic target for dry AMD.

## 1. Introduction

Age-related macular degeneration (AMD) is a progressive disease of central vision impairment, the leading cause of irreversible blindness in the elderly in developed countries [1]. Lipofuscinogenesis and early drusen genesis are early changes in AMD. Accumulation of lipid- and protein-rich molecules, known as drusen, which is a hallmark of the “early” dry clinical subtype of AMD, deposits along the basal surface of retinal pigment epithelium cells (RPE) and gradually results in RPE cell dysfunction and photoreceptor degeneration [2]. Epidemiological investigations have shown that several genetic variants associated with lipid biology are risk factors for advanced AMD [3,4].

RPE is extremely metabolically demanding and is rich in lipids. Over its lifespan, the RPE phagocytoses and degrades photoreceptor outer segments (POS), critical to normal photoreceptor function. Lipids accumulate in a high oxidative stress environment of macular during its course, oxidizing to oxysterol and further depositing to form drusen. Our previous study showed that 7-ketocholesterol (7KCh), deposited in the retina, causing retinal inflammation and dysfunction of RPE cell phagocytosis, thus leading to the occurrence and development of AMD [5]. Mitochondria are the power industry of cells and are significant in forming reactive oxygen species (ROS), energy, apoptosis (programmed cell death), and retrograde signaling. Significant changes in RPE mitochondria have been reported in AMD [6]. Mitophagy is a major process in the maintenance of mitochondrial homeostasis. Mitophagy is implicated in several neurodegenerative diseases [7,8], including AMD. Increased levels of ROS, excessive energy consumption, and DNA damage in RPE cells are all related to mitochondrial dysfunction and the pathogenesis of AMD [9].

Liver X receptors (LXRs) are transcriptional regulators that play an important role in cellular and systemic cholesterol homeostasis [10]. LXRα and LXRβ are two different isoforms of LXRs which are expressed in tissues and organs, including the retina and RPE cells [11]. LXRs have been shown to regulate the expression of a battery of lipid metabolic genes which contain those encoding ATP-binding cassette transporter A1 (ABCA1), apoE, and other apolipoproteins, increasing cholesterol efflux and promoting cholesterol reverse transport [12,13]. Considerable evidence has emerged proving that LXR activation boosts the expression of the ATP-binding cassette transporters A1 and G1 (ABCA1 and G1) and promotes cholesterol efflux in RPE cells [14]. Concerning the regulation of lipid metabolism, LXRs have been confirmed as physiological regulators of inflammation and phagocytosis [15,16,17]. Mitochondrial dysfunction has contributed to losing homeostasis and an imbalance in cellular sterol levels [18].

In the present study, we confirm that both LXRα and LXRβ are expressed in the retina. We found that oxysterols decreased mitochondrial membrane potential, thus inducing mitochondrial injury in ARPE-19 cells, while LXR agonists counteracted these effects of oxysterols. In cultured ARPE-19 cells, activating LXRs reduced p62, mTOR, and LC3I/II levels, and the knocking down of LXRs elevated the expression of proteins, indicating that activating LXRs could boost mitophagy. This study aimed to analyze the regulatory and protective effects of LXRs on lipid peroxide accumulation in the retina and elucidate its mechanism to provide more possibilities for the treatment of AMD.

## 2. Materials and Methods

### 2.1. Reagents

GW3965 (Selleck Chemicals Co., Huston, TX, USA) was dissolved in dimethyl sulfoxide (DMSO; Sigma-Aldrich Chemical Co., St. Louis, MO, USA) before use in cell culture and animal treatment. 7KCh and Oil Red O (ORO) staining was obtained from Sigma-Aldrich (St. Louis, MO, USA). Anti-LXRα, anti-LXRβ, anti-ABCA1, anti-p62, and anti-mTOR were purchased from Abcam (Cambridge, MA, USA). Fetal bovine serum (FBS) was obtained from Gibco (Thermo Fisher Scientific, Waltham, MA, USA). Lipofectamine 2000 was obtained from Invitrogen (Thermo Fisher Scientific, Waltham, MA, USA). The JC-1 Apoptosis Detection Kit was purchased from KeyGEN Biotech. Cell death was detected using an in situ cell detection kit (Roche Diagnostics Corp., Mannheim, Germany).

### 2.2. Animal Experiments

Six-week-old C57BL/6 mice were obtained from SPF (Beijing Biotechnology Co., Ltd., Beijing, China). All experimental procedures strictly conformed to the Association for Research in Vision and Ophthalmology Statement for the Use of Animals in Ophthalmic and Vision Research. All animals were raised in an SPF-level Laboratory Animal Room with a 12:12 h light/dark cycle at 24 ± 1 °C and 55–60% humidity. All animals had free access to standard food and drinking water. They were randomly divided into four groups (control, DMSO, 7KCh, and 7KCh+ GW3965 groups), with 10 mice (20 eyes) in each group. The control and 7KCh groups were intragastrically administered saline (100 μL/10 g), the DMSO group was intragastrically administered with 1% DMSO (100 μL/10 g), and the 7KCh+ GW3965 group was intragastrically administered GW3965 (dissolved in DMSO, 10 mg/kg according to the instructions) for three days continuously. On the fourth day, the mice in DMSO, 7KCh, and 7KCh+ GW3965 groups were subretinally injected with 1.5 µL 7KCh (20 μM) and then it was administered intragastrically for another four days.

### 2.3. Cell Culture and Transfection

The human retinal pigment epithelial cell line ARPE-19 (American Type Culture Collection, Manassas, VA, USA) cells were grown in Dulbecco’s Modified Eagle’s medium/F12 (DMEM/F12) supplemented with 10% FBS, 100 IU/mL penicillin, 2 mmol/L glutamine, and 100 μg/mL streptomycin in a humidified incubator with 5% CO_2_. The culture medium was updated every 48 h, and the cells of the fifth to eighth generations with good growth status were selected for subsequent experiments.

Cells were cultured in 6-well plates and divided into the control, DMSO, 7KCh, GW3965, siLXRα, and siLXRβ groups. In order to avoid the influence of drug toxicity on the cells, we chose concentration of 2μM GW3965 for cell culture (see Appendix A). The cells in group siLXRα/β were then transfected with siRNAs using Lipofectamine 2000. After transfection, the cells from different groups were treated with serum-free medium and 7KCh (20 μM), DMSO (0.02%, consistent with the concentration in the GW3965 group) or GW3965 (2 μM), respectively, for 24–48 h. The siRNA sequences targeting human LXRs were as follows: siLXRα-A, 5′-CCUCAAGGAUUUCAGUUAUTT-3′ (forward) and 5′-AUAACUGAAAUCCUUGAGGAA-3′ (reverse); siLXRα-B, 5′-GGAGUGUGUCCUGUCAGAATT-3′ (forward) and 5′-UUCUGACAGGACACACUCCTC-3′ (reverse); siLXRβ-B, 5′-CCAACUGCAGUGCAACAAATT-3′ (forward) and 5′-UUUGUUGCACUGCAGUUGGGC-3′ (reverse).

### 2.4. Cell Counting Kit-8 Assay

The ARPE-19 cell suspension was inoculated in 96-well plates (5 × 10^3^ cells/well) with a serum-free medium for 24 h. After 24 h, serum-starved cells were treated with different concentrations of GW3965 (1.5 µM, 2 µM, 2.5 µm, 3 µM) for 24 h. Afterward, CCK-8 (10 μL/well) was added to each well and further incubated at 37 °C for 2 h. After 2 h, the absorbance of each well was measured at 450 nm using a BIO-RAD microplate reader (Thermo, Waltham, MA, USA).

### 2.5. Immunofluorescence Staining

One day after gavage, immunofluorescence staining was performed to localize 7KCh. Briefly, enucleated eyes were immediately frozen in liquid nitrogen and then embedded in the OCT compound (Sakura, Torrance, CA, USA). The cryosections were cut along the optic nerve with 10 μm/piece. After drying the sections at room temperature (RT) for 30 min, they were incubated with 10% goat serum at RT for 2 h. After rinsing thrice in TBS (containing 0.025% Triton-X), the sections were incubated overnight with an anti-7KCh antibody (1:100) at 4 °C. Subsequently, the sections were stained with Cy3-labeled goat anti-mouse antibody (1:500) at RT for 2 h. DAPI was used to counterstain the nuclei.

After treatment, ARPE-19 cells were fixed in 4% paraformaldehyde for 20 min and washed with PBS three times. Immunofluorescence localization of 7KCh was conducted following the same procedure as previously described.

Images of mice retinas and ARPE-19 cells were captured using a confocal microscope (Leica, Wetzlar, Germany).

### 2.6. Oil Red O Staining of Retinas and ARPE-19 Cells

Cryosections were prepared and ORO staining (Sigma-Aldrich Chemical Co.) was performed to label the lipids. To prepare the ORO working solution, 0.5% ORO storage solution was diluted to 0.3% and sterilized by filtration. In brief, cryosections were washed with PBS twice and then stained in a freshly prepared ORO working solution for 20 min. The sections were then quickly rinsed with 60% isopropanol and PBS. Finally, the nuclei were counterstained with hematoxylin.

ARPE-19 cells were cultured and treated as previously described. The cells were rinsed three times with PBS and fixed in 4% paraformaldehyde for 20 min. ORO staining was performed as previously described.

ORO staining images of retina sections and ARPE-19 cells were captured using an upright microscope (Olympus BX53; Olympus, Tokyo, Japan).

### 2.7. Terminal Deoxynucleotidyl Transferase Mediated dUTP Nick end Labeling Assay of Mice Retinas

Terminal deoxynucleotidyl transferase mediated dUTP nick end labeling (TUNEL) assays were performed to observe the apoptosis of mice retina. First, cryosections were treated with protease K solution at RT for 20 min and rinsed with PBS twice. Secondly, samples were incubated with a TUNEL reaction mixture in the dark at 37 °C for 1h. After being washed with PBS three times, DAPI was added to stain the nuclei. The images were captured with a confocal microscope (Leica, Wetzlar, Germany) and the positive indensity was quantified with Image J software (National Institutes of Health).

### 2.8. Transmission Electron Microscope Examination

Transmission electron microscopy (TEM) was used to observe the ultrastructure. After removing the anterior segments, the eyeballs were fixed with 2.5% glutaraldehyde and added to 0.1 mol/L cacodylate buffer containing 0.2% tannic acid (PH 7.4), followed by washing twice in the same buffer. The central tissue that was 1mm around the optic nerve and 2 × 2 mm in size was postfixed with 1% osmium tetroxide. Tissue blocks were dehydrated, infiltrated and then embedded in Epon 812 resin. Para-phenylenediamine was added to protect the lipids. One micrometer-thick sections were firstly stained with uranyl acetate, followed by lead staining after washing. Images of the ultramicrostructure were captured by a Philips transmission electron microscope (CM120; Philips Tecnai, Eindhoven, The Netherlands).

### 2.9. Detection of Intracellular Reactive Oxygen Species (ROS)

After being treated with GW3965 (2 μM) and DMSO (0.02%) for 24 h, the cells in 35 mm Petri dishes were washed with PBS (PH 7.4) twice and then incubated with DCFH-DA at 37 °C for 30 min in the dark. After that, the cells were washed three times with a serum-free medium to remove the extracellular DCFH-DA. The fluorescence intensity was measured at an Ex./Em. = 488/525 nm of eight fields per dish. The ROS level was quantified by measuring fluorescence intensity with Image J software. The experiments were independently repeated three times. Results were shown as the mean value.

### 2.10. JC-1 Assay

The changes of mitochondrial membrane potential of the cells were detected by JC-1 staining. ARPE-19 cells were grown in black, clear-bottom 6-well plates. After treatment, the cells were washed with PBS three times and then blown into the cell suspension before adding JC-1 solution. The 1 × JC-1 working reagent was prepared by diluting 500 × JC-1 solution in the incubation buffer. The cells were incubated with a JC-1 working solution at 37 °C and 5% CO_2_ for 30 min. The cell suspension was centrifuged at 2000 rpm for 5 min at RT and washed twice with the incubation buffer to obtain a single-cell suspension. A flow cytometer (Beckman Coulter, Brea, CA, USA) was used to detect fluorescence (FL1, 488 nm; FL2, 530 nm).

### 2.11. Western Blot

ARPE-19 cells and mouse retina lysates were prepared with 4% sodium dodecyl sulfate (SDS). An equal amount of protein was resolved and separated by sodium dodecyl sulfate-polyacrylamide gel electrophoresis (SDS-PAGE), followed by transfer to polyvinylidene fluoride membranes (PVDF, Bio-Rad, Hercules, CA, USA). The membranes were blocked in 5% milk at 37 °C for 2 h and then blotted overnight at 4 °C with anti-ABCA1 (1:1000; Abcam), p62 (1:1000; Abcam), LC3 (1:1000; Abcam), mTOR (1:1000; Abcam), and β-actin (1:1000; Cell Signaling Technology, Danvers, MA, USA) antibodies separately. After that, the membranes were rinsed three times with TBST and incubated with anti-rabbit/mouse IgG (1:2000) at RT for 2 h. Signals were visualized by an enhanced chemiluminescence kit (GE Healthcare Life Sciences, Little Chalfont, UK). The experiments were independently repeated three times. Image J software was used to quantify the band intensities against β-actin control bands.

### 2.12. Statistical Analysis

All data were represented as the mean ± SD. The mean values of each group were analyzed by one-way ANOVA using GraphPad Prism software (version 5.0; GraphPad, San Diego, CA, USA). Test results with *p* < 0.05 were considered statistically significant.

## 3. Results

### 3.1. LXRs Activation in C57BL/6 Mice

**Figure 1:**
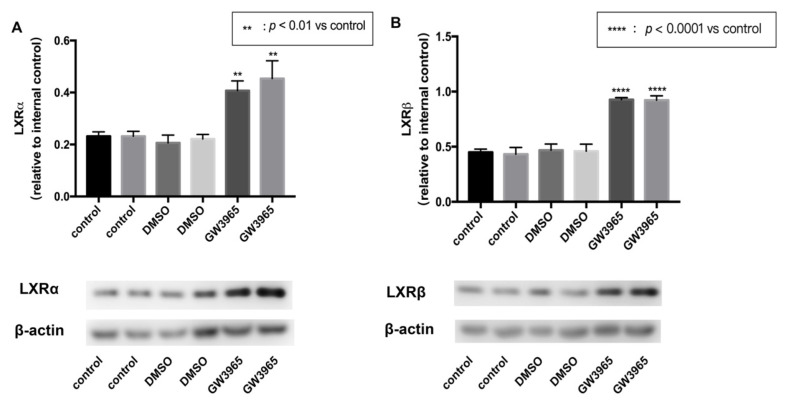
LXR activation in C57BL/6 mice. C57BL/6 mice were gavaged agonist (GW3965, 10 mg/kg) or vehicle (DMSO, 1%) continually for seven days. (Control) mice were gavaged saline for seven days (*n* = 3). (**A**) LXRα expression was determined by western blot and presented relative to controls. (**Β**) LXRβ expression was determined by western blot and presented relative to controls. Data are represented as mean ± SEM of three independent experiments. Differences of *p* < 0.05 were considered statistically significant.

To activate the expression of LXRs, we used an agonist GW3965. Mice were gavaged agonists or vehicles for seven days. After that, the eyeballs were removed, and the retina tissues were split separately. Total protein was extracted from the retinas using RIPA lysate. Western blot analysis showed that GW3965 activated LXRs, upregulating both LXRα and LXRβ (Figure 1A,B) in mice retinas.

### 3.2. 7KCh Immunofluorescence and Oil Red O Staining of Retinas

**Figure 2:**
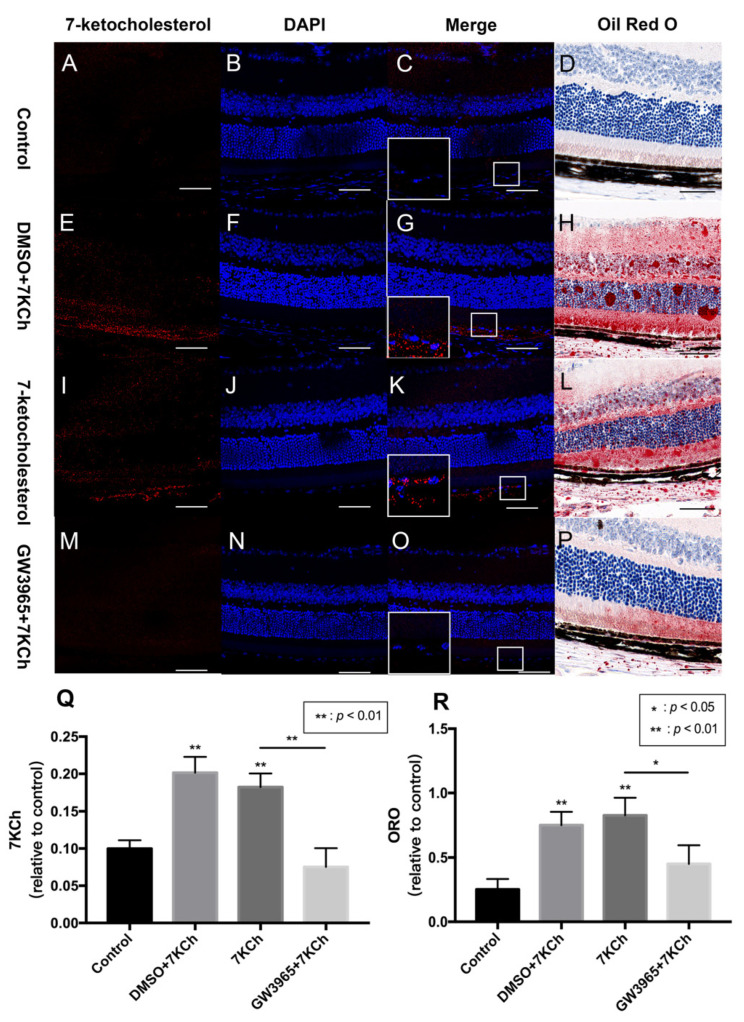
7KCh immunofluorescence and Oil Red O staining of retinas. 7KCh deposition was detected by immunofluorescence (**A**–**C**,**E**–**G**,**I**–**K**,**M**–**O**) and Oil Red O staining (**D**,**H**,**L**,**P**). (**A**–**D**) C57BL/6 mice were gavaged saline (100 μL/10 g) for seven days (*n* = 4). (**E**–**H**) Firstly, C57BL/6 mice were gavaged DMSO (1%) for three days, and then mice were subretinally injected 7KCh. After injection, gavaging with DMSO (1%) continued for four days. (**I**–**L**) C57BL/6 mice were gavaged saline (100 μL/10 g) for three days, then subretinally injected 7KCh. After injection, gavaging with saline (100 μL/10 g) continued for four days. (**M**–**P**) C57BL/6 mice were gavaged GW3965 (10 mg/kg) for three days, then the mice were subretinally injected 7KCh. After injection, gavaging with GW3965 (10 mg/kg) continued for four days. Retinas were harvested, sectioned, and subjected to double immunofluorescent staining and Oil Red O staining analyses. Scale bar 50 μm. (**Q**,**R**) 7KCh immunofluorescence and Oil Red O intensity were averaged at eight locations. Data are represented as mean ± SEM of three independent experiments. Differences of *p* < 0.05 were considered statistically significant.

Lipid metabolism is considered an important event in AMD; therefore, we investigated the ability of the RPE cells to regulate lipid accumulation in choriocapillaris-BrM-RPE cells. We performed a subretinal injection of 7KCh in C57BL/6 mice and detected the presence of 7KCh deposition in RPE cells and subretinal areas. Immunofluorescence results showed that 7KCh staining in control and GW groups was negative, while 7KCh staining was found in the DMSO and 7KCh groups, indicating that gavaging with GW3965 reduced 7KCh deposition in the retina (Figure 2C,G,K,O,Q). Oil red O staining revealed no significant lipid deposition in the retina of mice in the control group (Figure 2D), while a large number of lipid droplets were observed in the DMSO and 7KCh groups (Figure 2H,L). Compared with those in the DMSO and 7KCh groups, the lipid droplets in the retina of mice in the GW3965 group reduced significantly (Figure 2P), indicating that the agonist reduced lipid deposition between retinal layers (Figure 2R).

### 3.3. Apoptosis of RPE Cells Detected by TUNEL Assay

**Figure 3:**
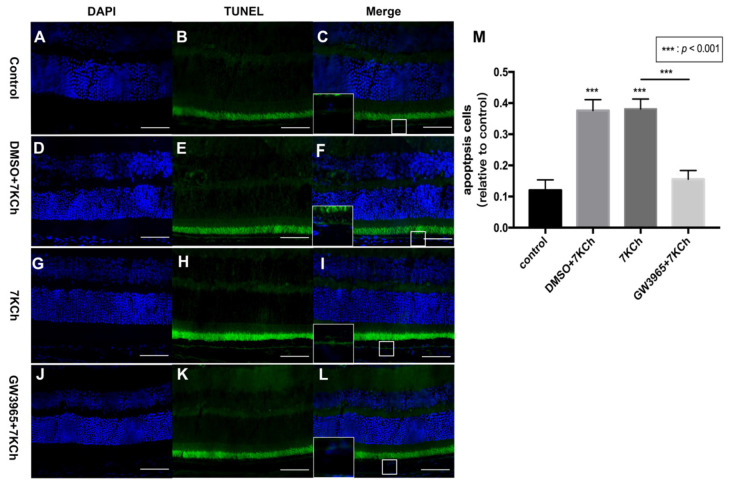
Apoptosis of RPE cells detected by TUNEL assay. (**A**–**C**) C57BL/6 mice were gavaged saline (100 μL/10 g) for seven days (*n* = 4). (**D**–**F**) Firstly, C57BL/6 mice were gavaged DMSO (1%) for three days, and then they were subretinally injected 7KCh. After injection, gavaging with DMSO (1%) continued for four days. (**G**–**I**) C57BL/6 mice were gavaged saline (100 μL/10 g) for three days, then they were subretinal injected 7KCh. After injection, gavaging with saline (100 μL/10 g) continued for four days. (**J**–**L**) C57BL/6 mice were gavaged GW3965 (10 mg/kg) for three days, and then they were subretinally injected 7KCh. After injection, gavaging with GW3965 (10 mg/kg) continued for four days. Retinas were harvested, sectioned, and subjected to TUNEL analyses. Blue represents nuclei, and the green represents apoptosis cells. Scale bar 50 μm. (**M**) Fluorescent intensity was measured at eight different locations and averaged. Data are represented as mean ± SEM of three independent experiments. Differences of *p* < 0.05 were considered statistically significant.

The TUNEL staining of mouse retinas showed that there were fewer positive cells in the control group (Figure 3C), but more positive cells in the DMSO and 7KCh groups (Figure 3F,I), indicating that 7KCh induced apoptosis in the retinal RPE cell layer of mice (Figure 3M). Fewer positive particles were found in the GW3965 group than in the 7KCh group (Figure 3I,L), indicating that activating LXRs could reduce RPE cell apoptosis (Figure 3M).

### 3.4. Ultrastructural Observations

**Figure 4:**
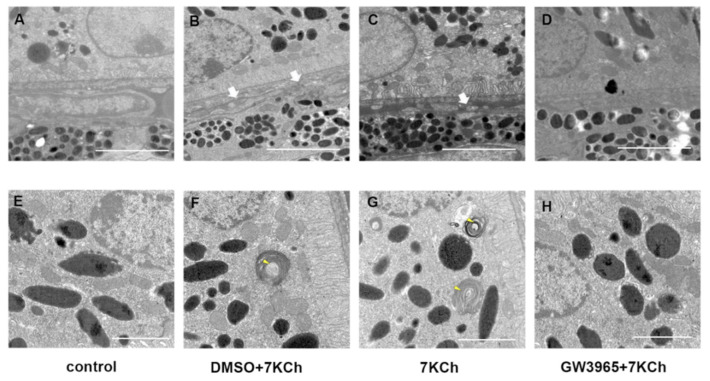
TEM examination of the retinas. (**A**,**E**) C57BL/6 mice were gavaged saline (100 μL/10 g) for seven days. (**B**,**F**) First, C57BL/6 mice were gavaged DMSO (1%) for three days, and then they were subretinally injected 7KCh. After injection, gavaging with DMSO (1%) continued for four days. (**C**,**G**) C57BL/6 mice were gavaged saline (100 μL/10 g) for three days, then they were subretinally injected 7KCh. After injection, gavaging with saline (100 μL/10 g) continued for four days. (**D**,**H**) C57BL/6 mice were gavaged GW3965 (10 mg/kg) for three days, and then they were subretinally injected 7KCh. After injection, gavaging with GW3965 (10 mg/kg) continued for four days (*n* = 3). White arrows indicate electron-dense material above the BrM. Yellow arrows indicate undigested POS. (**A**–**D**), scale bar 2 μm. (**E**,**F**), scale bar 1 μm.

Figure 4A shows a normal RPE cell and Bruch membrane: well-organized basal infolding, no vacuoles nor sub-RPE deposits. We identified degenerative changes in the RPE of C57BL/6 mice in 7KCh and DMSO groups, including basement electron-dense sediments above the BrM and disorganized basal infolding (Figure 4B,C). Abnormal photoreceptor segments were also found in mice from the 7KCh and DMSO group (Figure 4F,G). In contrast, RPE/BrM of LXR-activated mice organized regularly without any change (Figure 4D,H).

### 3.5. 7KCh Immunocytofluorescence and Oil Red O Staining of ARPE-19 Cells

**Figure 5:**
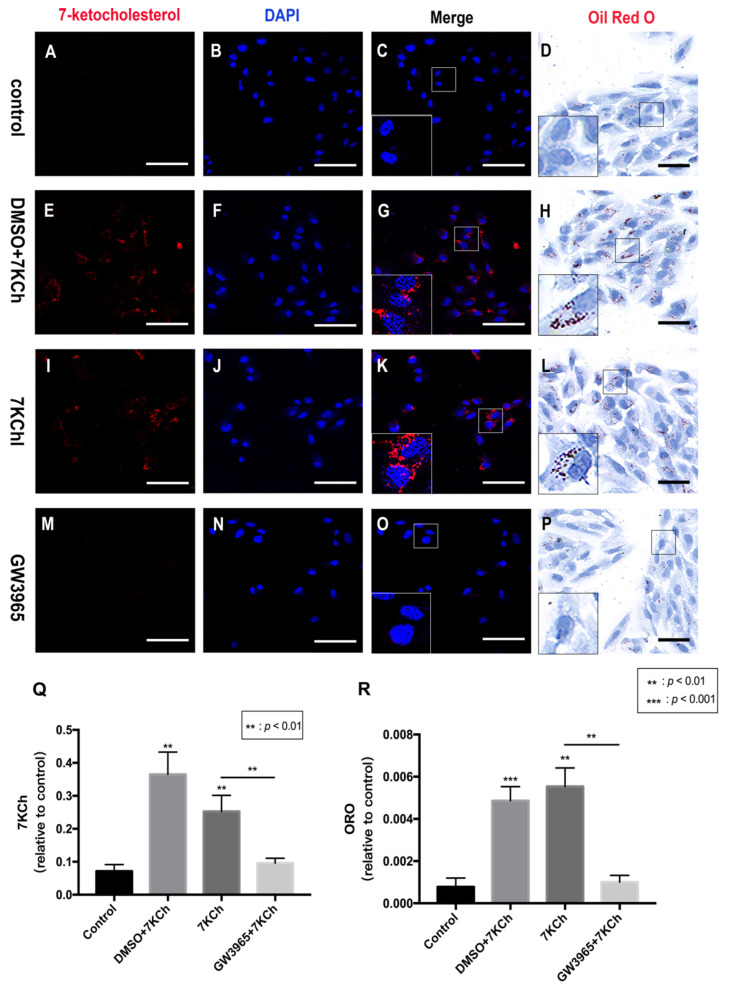
7KCh immunocytofluorescence and Oil Red O staining of ARPE-19 cells. 7KCh deposition was detected by immunofluorescence (**A**–**C**,**E**–**G**,**I**–**K**,**M**–**O**) and Oil Red O staining (**D**,**H**,**L**,**P**). (**A**–**D**). As a control, ARPE-19 cells were cultured with a serum-free medium for 48 h. (**E**–**H**) ARPE-19 cells were incubated with DMSO (0.02%) for 24 h following 7KCh (20 μM). (**I**–**L**) ARPE-19 cells were cultured with serum-free medium for 24 h and then incubated with 7KCh (20 μM) for 24 h. (**M**–**P**) ARPE-19 cells were incubated with GW3965 (2 μM) for 24 h following 7KCh (20 μM). The cells were harvested and subjected to double immunofluorescent staining and Oil Red O staining analyses. White scale bar 75 μm. Black scale bar 50 μm. (**Q**,**R**) 7KCh immunofluorescence and Oil Red O intensity were averaged at eight locations. Data are represented as mean ± SEM of three independent experiments. Differences with *p* < 0.05 were considered statistically significant.

Immunofluorescence and ORO staining showed that activating LXRs can reduce lipid accumulation in mice retinas. Immunocytofluorescence staining of ARPE-19 cells showed that 7KCh-positive cells in the 7KCh group were higher than that in the control (Figure 5C,K), indicating that 7KCh was deposited in RPE cells. Cells treated with GW3965 showed decreased intracellular positive particles compared with the control group treated with DMSO, indicating decreased 7KCh accumulation in RPE cells (Figure 5G,O,Q). ORO staining also confirmed fewer intracellular lipid drops in the control group than in the 7KCh group (Figure 5D,L). No significant difference was observed between the intracellular lipid drops in the DMSO group and those in the 7KCh group (Figure 5H,L). In addition, the intracellular lipid drops in the GW3965 group were lower than those in the 7KCh and DMSO groups (Figure 5 H,L,P). In other words, LXR agonist treatment reduced the accumulation of 7KCh in RPE cells (Figure 5R).

### 3.6. Apoptosis of ARPE-19 Cells Measured by ROS Level

**Figure 6:**
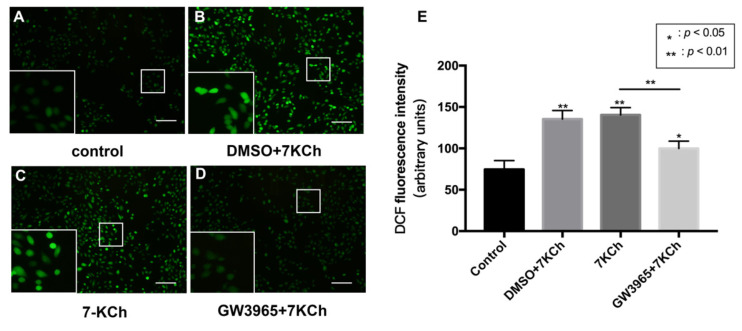
ROS levels of ARPE-19 cells. (**A**) As a control, ARPE-19 cells were cultured with a serum-free medium for 48 h. (**B**) ARPE-19 cells were incubated with DMSO (0.02%) for 24 h, followed by 7KCh (20 μM) for 24 h. (**C**) ARPE-19 cells were cultured with serum-free medium for 24 h and then incubated with 7KCh (20 μM) for 24 h. (**D**) ARPE-19 cells were incubated with GW3965 (2 μM) for 24 h, followed by 7KCh (20 μM) for 24 h. The apoptosis of ARPE-19 cells was measured by ROS detection. Scale bar 100 μm. (**E**) DCF fluorescence intensity was quantified with Image J. Data are represented as mean ± SEM of three independent experiments. Differences with *p* < 0.05 were considered statistically significant.

In the TUNEL assay, we observed that activating LXRs reduced apoptosis cells in the retina. We hypothesized that LXRs might prevent RPE cell apoptosis after 7KCh treatment. The apoptosis of ARPE-19 cells was measured by ROS detection (Figure 6). The total ROS level was significantly increased after 7KCh exposure (Figure 6A–C), and GW3965 counteracted the effect, suggesting that activating LXRs may protect RPE cells from apoptosis.

### 3.7. LXRs Activation in ARPE-19 Cells

**Figure 7:**
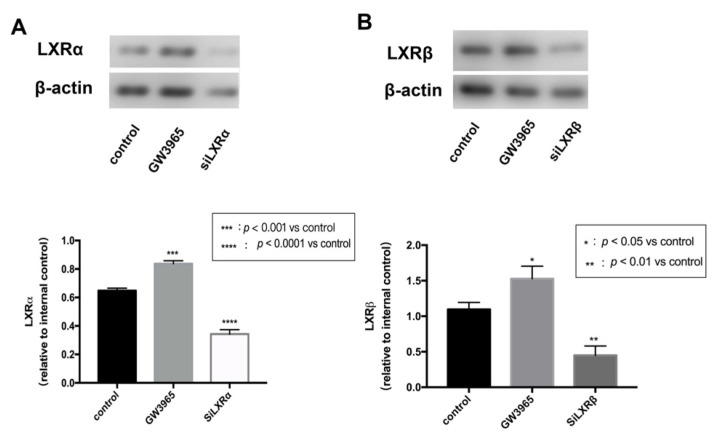
LXR activation in ARPE-19 cells. (**A**) LXRα expressions were detected by western blot and presented relative to controls. (**Β**) LXRβ expressions were detected by western blot and presented relative to controls. Data are represented as mean ± SEM of three independent experiments. Differences with *p* < 0.05 were considered statistically significant.

To investigate the regulatory effects of LXRs in RPE cells, we measured LXRs expression in ARPE-19 cells after GW3965 treatment and LXRs knockdown. ARPE-19 cells were treated with agonists or siRNAs for 24 h. Western blot analysis of protein collected from ARPE-19 cells showed that GW3965 activated LXRs, upregulating both LXRα and LXRβ (Figure 7A,B) in ARPW-19 cells. The transfection efficiency was also confirmed by western blot (Figure 7A,B).

### 3.8. Flow Cytometric Analysis of Mitochondrial Membrane Potential in ARPE-19 Cells

**Figure 8:**
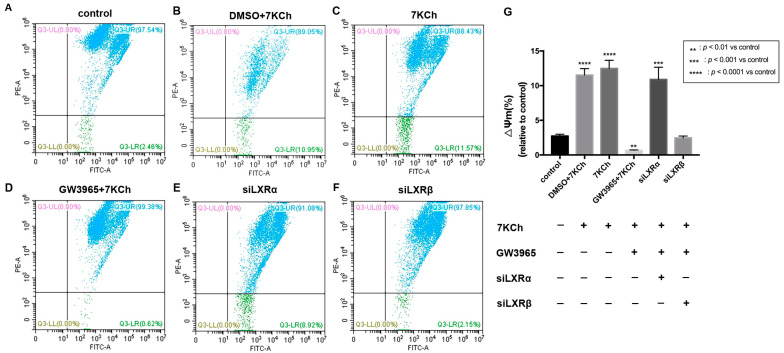
Flow cytometric analysis of mitochondrial membrane potential in ARPE-19 cells. (**A**) As a control, ARPE-19 cells were cultured with a serum-free medium for 48 h. (**B**) ARPE-19 cells were incubated with DMSO (0.02%) for 24 h, followed by 7KCh (20 μM) for 24 h. (**C**) ARPE-19 cells were cultured with serum-free medium for 24 h and then incubated with 7KCh (20 μM) for 24 h. (**D**) ARPE-19 cells were incubated with GW3965 (2 μM) for 24 h, followed by 7KCh (20 μM) for 24 h. (**E**) ARPE-19 cells were first transfected with siLXRα as previously described and then incubated with 7KCh (20 μM) and GW3965 (2 μM) for 24 h. (**F**) ARPE-19 cells were first transfected with siLXRβ as previously described and then incubated with 7KCh (20 μM) and GW3965 (2 μM) for 24 h. After treatment, the cells were collected and subjected to flow cytometric analysis of mitochondrial membrane potential. (**G**) Mitochondrial membrane potential was quantified. Data are represented as mean ± SEM of three independent experiments. Differences of *p* < 0.05 were considered statistically significant.

JC-1 detection of the mitochondrial membrane potential of ARPE-19 cells showed that apoptotic cells accounted for 2.46% of normal ARPE-19 cells (Figure 8A). After 7 KCh treatment, the percentage of apoptotic cells in the 7KCh group increased by 9.11% compared with that in the normal control group (*p* < 0.0001) (Figure 8A,C). Following the treatment with GW3965 (2 μΜ) and 7KCh (20 μM), the percentage of apoptotic cells was 0.62%, which was 1.84% less than the normal control group (*p* < 0.05) (Figure 8A,D). GW3965 can maintain the electrical depolarization and potential balance of the mitochondrial membrane, thereby protecting the stability of mitochondrial function (Figure 8G). After the knockdown of LXRα, the proportion of apoptotic cells increased, indicating that siLXRα partially offset the protective effect of GW3965-activated LXRs on ARPE-19 cells (Figure 8E); however, no such result was observed following the knockdown of LXRβ (Figure 8F), indicating that LXRα plays a major role in maintaining the stability of mitochondrial membrane potential between the two types of LXRs.

### 3.9. Western Blot Analysis of Protein in ARPE-19 Cells

**Figure 9:**
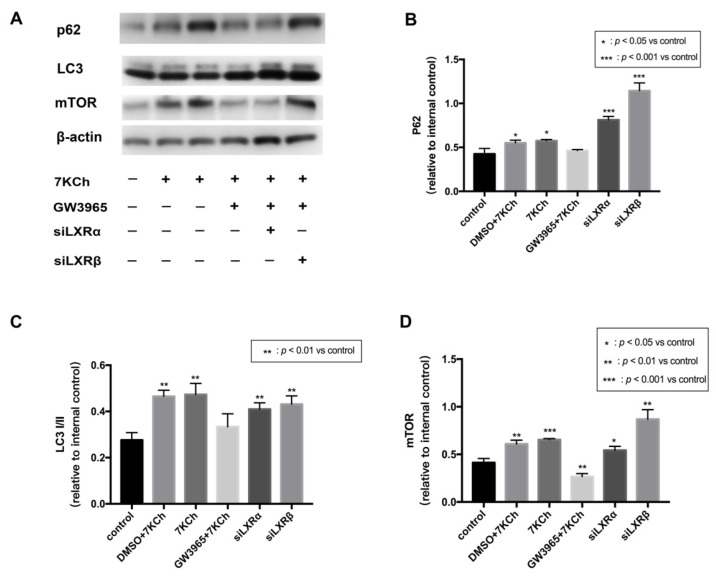
Western blot of proteins in ARPE-19 cells. (**A**) As a control, ARPE-19 cells were cultured with a serum-free medium for 48 h. (DMSO) ARPE-19 cells were incubated with DMSO (0.02%) for 24 h, followed by 7KCh (20 μM) for 24 h. (7KCh) ARPE-19 cells were cultured with serum-free medium for 24 h and then incubated with 7KCh (20 μM) for 24 h. (GW3965) ARPE-19 cells were incubated with GW3965 (2 μM) for 24 h, followed by 7KCh (20 μM) for 24 h. (siLXRα) ARPE-19 cells were first transfected with siLXRα as previously described and then incubated with 7KCh (20 μM) and GW3965 (2 μM) for 24 h. (siLXRβ) ARPE-19 cells were first transfected with siLXRβ as previously described and then incubated with 7KCh (20 μM) and GW3965 (2 μM) for 24 h. Total protein was collected from the cultured ARPE-19 cells. (**B**) P62 expression levels in ARPE-19 cells. (**C**) LC3I/II expression levels in ARPE-19 cells. (**D**) mTOR expression levels in ARPE-19 cells. Data are represented as mean ± SEM of three independent experiments. Differences with *p* < 0.05 were considered statistically significant.

The loss of mitochondrial membrane potential (MMP) is a marker of mitophagy [19], and LXR and autophagy can reciprocally activate each other [20]. JC-1 analysis revealed that LXRs protected ARPE-19 cells from apoptosis by maintaining mitochondrial membrane stasis. Thereby we hypothesize that LXRs could regulate mitophagy. p62 is known to be one of the mitophagy markers [21]. LC3 is one of Atg8p (autophagy-related) homologs that regulate selective autophagy [22]. mTOR is known to be major regulator which could inhibit autophagy [23]. We measured the levels of the mitophagy receptor p62 and its corresponding proteins. mTOR, p62, and LC3I/II were all upregulated in ARPE-19 cells by 7KCh treatment, indicating that mitophagy was blocked after 7KCh treatment (Figure 9A–D). In the GW3965 group, the LXR agonist promoted mitophagy by decreasing mTOR, p62, and LC3I/II levels in ARPE-19 cells; however, the effect could be inhibited by the knockdown of LXR.

## 4. Discussion

Lipid metabolism dysfunction is closely related to the pathogenesis of AMD [24]. Drusen has been identified to have rich lipid deposits and oxidation products [25]. Lipid metabolism is subtly regulated in the retina, and its dysregulation can lead to lipid accumulation in RPE cells and Bruch’s membrane [26]. Studies have shown that lipid metabolism disorders cause lipid accumulation in RPE cells and Bruch’s membrane, which is then oxidized to oxysterols under the highly oxidative environment of the macula and gradually deposited to form drusen [27,28]. RPE cells play an important role in regulating lipid metabolism in the eyes [29]. Our previous study displayed that RPE cells could take up 7 KCh initially and induce inflammation through the ERK signaling pathway [5]. This study performed a subretinal injection to deliver 7 KCh to the interphotoreceptor/subretinal compartment [30]. C57BL/6 mice were treated with GW3965 for a week to decrease the expression of LXR. Consistent with our hypothesis, the activation of LXRs accelerated lipid metabolism and reduced lipid deposits in the retina. On top of this, we observed that 7KCh caused RPE degeneration, while few changes were documented in LXR-activated retinas under TEM. TUNEL examination showed increased RPE cell apoptosis after 7 KCh injection and decreased RPE cell apoptosis in GW3965 group mice. In vivo experiments confirmed that LXRs regulate lipid metabolism in RPE cells and protect RPE cells from apoptosis. Hence, we investigated the protective mechanism of LXRs in vitro.

The phagocytosis of RPE cells is highly active and requires a large amount of energy. Healthy mitochondria are the foundation of energy production. The mitochondria stability is significant for the phagocytosis and homeostasis of RPE cells. The mitochondrial regulation of cholesterol homeostasis plays an important role in lipid metabolism18. Additionally, oxysterols can harm mitochondria. 7-oxysterols induce the induction and nuclear translocation of p53 in M1-t-p53 cells, enhancing LMP, mitochondrial translocation of Bax, mitochondrial membrane permeabilization, and the cytosolic release of cytochrome c, and cell death [31]. 7KCh has been shown to increase ROS levels in human neurocytes [32] rapidly. In addition, miRNA-1 was found to regulate the LXRα-ROS-mitochondrial pathway to regulate cardiomyocyte apoptosis [33]. Therefore, we measured the mitochondrial potential of the ARPE-19 cells. The results show that LXRs protected ARPE-19 cells from apoptosis by maintaining mitochondrial membrane stasis.

Autophagy is an important declaration that maintains cell homeostasis. Under different conditions, autophagy may occur selectively, such as mitophagy and ER-phagy. The role of autophagy disorders in a range of neurodegenerative diseases, including AMD, has been widely confirmed [34,35,36]. LXRs can interact with autophagy and reduce atherosclerosis susceptibility [20]. We found that LXR could protect mitochondria in RPE cells; therefore, we wondered whether LXR could regulate mitophagy. Western blot analysis revealed that LXR decreased mitophagy receptor p62 and LC3I/II levels and mTOR secretion, indicating increased mitophagy flow and activation of mitochondrial autophagy in ARPE-19 cells. We demonstrated that LXRα and LXRβ jointly activate mitochondrial autophagy through the mTOR/p62 pathway and promote the mitochondrial renewal metabolism.

In conclusion, LXRs activation improves the oxidized lipids damage in RPE cells by expediting lipid metabolism. LXRα and LXRβ jointly activate mitochondrial autophagy in the mTOR/p62 pathway, protecting mitochondrial renewal and normal function from peroxidation damage. With the evidence above, we believe that LXR can become a new target for treating dry AMD in the future.

## Figures and Tables

**Figure A1:**
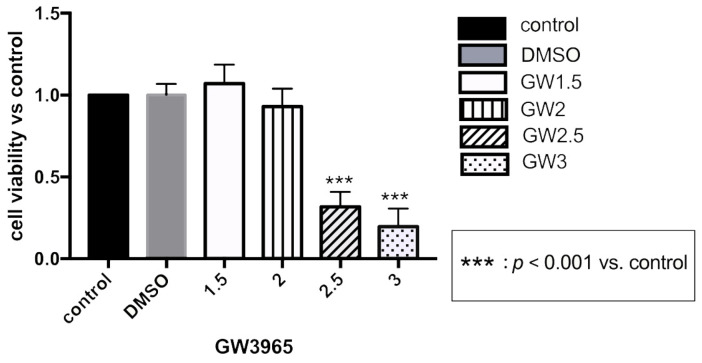
Assay after GW3965 treatment in ARPE-19 cells. ARPE-19 cells were cultured with DMSO, different concentrations of GW3965 or nothing in serum-free medium for 48 h. Cell viability was measured and quantified. Data are represented as mean ± SEM of three independent experiments. Differences with *p* < 0.05 were considered statistically significant.

## Data Availability

The data presented in this study are available on request from the corresponding author.

## Appendix A

### CCK-8 Assay for Evaluation of Cell Viability after GW3965 Treatment

We used the CCK-8 assay to detect the effect of GW3965 on the viability of ARPE-19 cells to determine the treatment concentration. The results showed that ARPE-19 cell viability was inhibited at GW3965 concentrations of 2.5 μM or higher; therefore, we used a concentration of 2 μM for ARPE-19 cell treatment.
